# The DIY Digital Medical Centre

**DOI:** 10.1111/1751-7915.12817

**Published:** 2017-08-25

**Authors:** James Kenneth Timmis, Kenneth Timmis

**Affiliations:** ^1^ Student MSc Health Policy Department of Surgery and Cancer Imperial College London London UK; ^2^ Department of Microbiology Technical University of Braunschweig Braunschweig Germany

## Abstract

Healthcare systems worldwide are confronted with major economic, organizational and logistical challenges. Historic evolution of health care has led to significant healthcare sector fragmentation, resulting in systemic inefficiencies and suboptimal resource exploitation. To attain a sustainable healthcare model, fundamental, system‐wide improvements that effectively network, and ensure fulfilment of potential synergies between sectors, and include and facilitate coherent strategic planning and organisation of healthcare infrastructure are needed. Critically, they must be specifically designed to sustainably achieve peak performance within the current policy environment for cost‐control, and efficiency and quality improvement for service delivery. We propose creation of a new healthcare cluster, to be embedded in existing healthcare systems. It consists of (i) local 24/7 walk‐in virtually autonomous do‐it‐yourself Digital Medical Centres performing routine diagnosis, monitoring, prevention, treatment and standardized documentation and health outcome assessment/reporting, which are online interfaced with (ii) regional 24/7 eClinician Centres providing on‐demand clinical supervision/assistance to Digital Medical Centre patients. Both of these are, in turn, online interfaced with (iii) the National Clinical Informatics Centre, which houses the national patient data centre (cloud) and data analysis units that conduct patient‐ and population‐level, personalized and predictive(‐medicine) intervention optimization analyses. The National Clinical Informatics Centre also interfaces with biomedical research and prioritizes and accelerates the translation of new discoveries into clinical practice. The associated Health Policy Innovation and Evaluation Centre rapidly integrates new findings with health policy/regulatory discussions. This new cluster would synergistically link all health system components in a circular format, enable not only access by all arms of the health service to latest patient data, but also automatic algorithm analysis and prediction of clinical development of individual patients, reduce bureaucratic burden on medical professionals by enabling a greater level of focus of their expertise on non‐routine medical tasks, lead to automatic translation of aggregate patient data/new knowledge into medical practice, and orient future evolution of health systems towards greater cohesion/integration and hence efficiency. A central plank of the proposed concept is increased emphasis on reduction of disease incidence and severity, to diminish both patient suffering and treatment costs. This will be achieved at the individual and population levels, through (i) significantly improved access to medical services, (ii) stronger focus on primary and secondary prevention and early treatment measures, and disease susceptibility prediction via personalized medicine, involving *inter alia* genome analysis at birth and periodic analysis of microbiomes and biomarkers, and integration with other patient health and epidemiology parameters, (iii) improved surveillance and (iv) intervention outcome benchmarking. The dMCs will become drivers of innovation and integrative evolution in health systems, of disease reduction and efficiency gains, and thus major contributors to development of sustainability of health care.

## Introduction

Over the past century, spectacular advances in medicine have resulted in previously unimaginable improvements in individual and public health. Historically, the evolution of medicine has been largely organic, driven by new discoveries, developments and opportunities, rather than through concerted strategic efforts to maximize synergetic effects, efficiency and satisfy long‐term future priority needs. This has led to significant fragmentation of clinical expertise, healthcare responsibilities, patient data, financing and infrastructure, policy development and governance, etc., and thus collectively provide for major potential to improve efficiency and cohesion (see e.g. Hirsch *et al*., 2016; Stange, 2009). In addition, diverse factors, such as increases in life expectancy and average income, health system configuration, (Chernew and Newhouse, 2011), etc., have substantially contributed to unsustainable rates of expenditure increases for what is arguably our most valued public service. As healthcare resources are limited, improving healthcare systems’ performance, i.e. efficiency, is considered the only viable long‐term policy option towards their financial sustainability. Although many high‐income countries have, over the past 3 decades, introduced policies to in many cases successfully control, i.e. contain and reduce, costs and incentivize increased efficiency, quality of healthcare delivery and improved access for patients^1^, healthcare costs continue to rise, and their financial sustainability in high‐income countries, and affordability to establish (and improve) adequate coverage in low‐ and middle‐income countries, remain important issues (see e.g. Jamison *et al*., 2013; OECD, 2015a,b). We and others (see e.g. National Information Board and Department of Health, 2014; NHS England, 2017; Select Committee on the Long‐term Sustainability of the NHS, 2017) contend that system‐wide defragmentation, and establishment of cohesive and effective healthcare service life cycle development, planning, delivery and management procedures, are urgently required for significant progress towards affordability and financial sustainability of healthcare systems. However, as far as we are aware, existing reports and analyses for improving health care in diverse functions and settings tend to focus on solving individual shortcomings or more abstract aims, but fail to detail concrete and comprehensive concepts/roadmaps to synergistically integrate system‐wide efforts, and thus to change the current paradigm of healthcare innovation and translational medicine, delivery design, coordination and implementation. In particular, full and coordinated exploitation of new healthcare technologies, the significance of which in improving health outcomes has been documented (inter alia by Jamison *et al*., 2013), and of microbial biotechnology (see some examples of highly relevant research and innovations in this issue), are in our opinion key facilitators to significantly and sustainably enhance performance across the entire healthcare sector and, importantly, at the point of access.

## Towards a solution

To significantly improve the quality and effectiveness of healthcare delivery, we contend that future evolution must be guided by strategic planning of the entire system for coherence, rather than of its component parts (see e.g. NHS England, 2017). Significantly greater emphasis must be placed on purpose‐focused use of healthcare resources, i.e. maximizing utilization of services according to their intended purpose, e.g. physicians for tasks requiring medical training, not administration, etc., by consistently and comprehensively exploiting rapid implementation of newest autonomous technological and informational advances, reducing physician–patient burden, increasing patient participation in diagnosis, monitoring, prevention and treatment procedures, and replacing linearity with circularity in the flow and analysis of patient data. To this end, we propose creation of a new three‐component healthcare cluster, seamlessly integrated and intelligently networked, to be incorporated in existing healthcare infrastructure.

**Figure 1:**
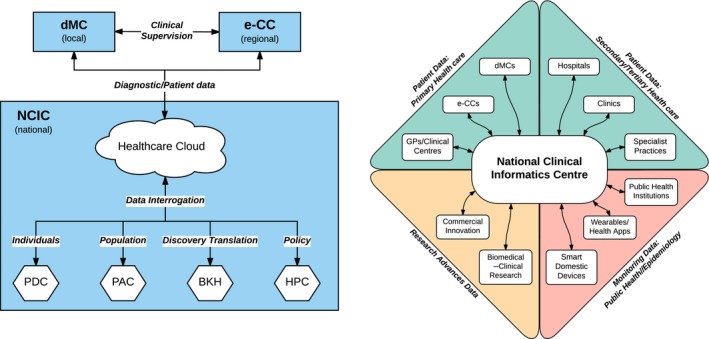
The new healthcare cluster. Left‐hand panel: new healthcare cluster configuration; right‐hand panel: The Healthcare Data–Biomedical Knowledge Highway Network.

The new triad (Fig. 1, left‐hand panel) will consist of (i) local 24/7 Digital Medical Centres (dMCs) based on smart, automated, patient interrogation/diagnostic machines, constantly interfaced online with both (ii) regional eClinician Centres (eCCs) functioning 24/7 and (iii) a central National Clinical Informatics Centre (NCIC).

The healthcare cluster proposed here is underpinned by four key principles:

- greater emphasis on disease reduction – primary/secondary prevention, and predictive medicine – requiring *inter alia*, quasi‐universal genome determination (preferentially determined at birth), and regular clinically relevant biomarker determination and microbiome analysis,
- creation of, or substantive improvement of an existing, standardized, coevolving digital healthcare patient documentation system designed for comprehensive and efficient handling, interrogation and comparison of individual and aggregate clinically relevant data, and for systematically and sustainably improving intervention outcomes (closing the information circle and supporting artificial intelligence learning),
- greater participation of an increasingly medically literate proactive patient in health procedures,
- optimal alignment of medical, commercial and regulatory strategic goals for incentivizing development of low‐cost generic and novel high‐benefit products (see e.g. Naci *et al*., 2015, for issues arising from misaligned incentives for pharmaceuticals) that address strategic health priorities, and increased commitment of the health system to such products.

## The components of the new healthcare cluster

### The do‐it‐yourself Digital Medical Centres (dMCs)

The dMCs, see also (Timmis and Timmis, 2017), are 24/7, local, walk‐in, virtually autonomous, clinically designed and supervised environments, featuring advanced but easy‐to‐use intelligent interrogation/diagnostic machines, facilitating determination of *inter alia* physical, cognitive and mental health parameters. The smart machines (primary patient interface) are permanently interfaced with remote, regional, 24/7 eClinician Centres and the Healthcare Cloud (HC, see below). dMCs initially assume following tasks:

i Patient interrogation: HC‐linked smart machines query relevant parameters of general nature, e.g. for annual check‐ups, and syndrome‐specific, e.g. assessment of new complaints and/or minor injuries, follow‐up after hospital discharge, and monitoring existing and/or chronic conditions (remotely clinician‐guided where necessary).
ii Perform a range of basic to sophisticated diagnostic procedures, including weight–height determination, blood pressure/pulse rate measurement, physical inspection, acquisition of clinically relevant images (e.g. of wounds, dermatological features), cognition assessment. Patients are instructed to provide/take clinical samples (e.g. breath, sweat, saliva, urine, blood, microbiome), where necessary assisted by on‐site nurses. Samples are analysed on‐site or offsite, e.g. dMC machines dispense and analyse a range of lateral flow diagnostic assay strips and immediately take the results into consideration and, in parallel, upload them to the HC. Other, dedicated, smart rooms contain more sophisticated (patient‐instructing) diagnostic and imaging (see e.g. Curiel‐Lewandrowski, 2016; Dengel *et al*., 2015) machines. The range of parameters obtainable continuously expands with the development of increasingly user‐friendly, more compact diagnostic machines.
iii Diagnosis/therapy, involving probability determination by advanced algorithms indicating with clinically sufficient confidence:
  - no treatment indicated, e.g. for mild ailments or minor injuries,
  - prescription of
    - prophylactic/therapeutic measures from the on‐site pharmacy/nurse (e.g. chronic conditions, minor injuries), etc.,
    - follow‐up clinical parameter (OECD, 2015a) monitoring, at dMCs or clinical centres, or at home, e.g. wearable devices, mobile applications for devices equipped with appropriate sensors, smart domestic appliances.
iv Immediate video interfacing with eClinicians when diagnoses with clinically insufficient confidence are attained, serious conditions determined, or patients feel the need
v Stock medications, vaccines, etc. (and, in future, synthesize personalized vaccines, medications, print medical equipment and devices via 3D printers, etc., on‐demand)
vi Follow‐up: determination and recording of intervention outcome effectiveness

### eClinician Centres (eCCs)

Remote eClinicians (general practitioners and mental health professionals with extensive knowledge of the diagnostic capabilities of the dMC smart machines and their interrogation algorithms) located in regional eClinician Centres functioning 24/7 are the secondary interface for dMC patients. eClinicians have direct access to the Healthcare Cloud records and the newly determined dMC data. Their involvement with dMC patients is needs‐based, triggered by the patient or diagnostic results obtained by dMC smart machines that flow in real time to the eCCs and are automatically analysed by advanced and continuously evolving algorithms, which alert the on‐duty clinicians to any need for involvement. eClinicians initiate additional tests for suspected conditions at the dMCs, reach diagnosis and prescribe a treatment, or make referrals to specialists. A national eCC is charged with prompt handling of indicated potential public health threats.

The dMC‐eCC tandem of 24/7 clinical diagnostic therapeutic care services will reduce bottlenecks at patient point of access, providing virtually immediate care for routine services (and promote patient autonomy/awareness for health‐preserving/healthcare‐cost‐saving behaviour), and contribute to a reduction in disease burden and patient discomfort. This will, in turn, rebalance and focus primary health services’ workloads and expertise: physicians will be able to concentrate on tasks requiring their advanced training, complement their services through access to comprehensive personalized patient data sets and latest intervention recommendations (see below), and will benefit from a reduction of routine documentation and administrative tasks.

### National Clinical Informatics Centre (NCIC)

The NCIC is the national healthcare information and analysis centre housing the Healthcare Cloud. It constitutes the framework of a smart, cognitive artificial intelligence/machine‐learning supported (see e.g. Chen *et al*., 2016; SAP, 2016) clinical knowledge hub. The NCIC is responsible for handling all patient data and multiperspective (individual, aggregate, etc.; disease‐, symptom‐, molecule‐, active ingredient‐specific, etc.; stratum‐, gene‐specific, etc.) data analysis and, *inter alia*, outcome effectiveness indicator determination. It focuses on disease diagnosis, treatment, prevention and prediction strategies, prioritization of efficacy and effectiveness trials, steering implementation of new procedures and health technology developments according to existing and prospective clinical needs, and hence optimization of healthcare resources. It interfaces with biomedical and health policy research and translates trends, analyses and validated clinically relevant advances into proposals for clinical practice and health policy. It consists of five integrated components.

#### Healthcare Cloud (HC)

The Healthcare Cloud is the patient data warehouse that collects and stores in a standardized and secure format all clinically relevant data received from multiple entities, such as dMCs and other primary, secondary and tertiary healthcare providers, biomedical research institutions, public health authorities and smart monitoring devices (see Fig. 1 and, for an example of HC proposal, Bahga and Madisetti, 2013). It also contains the specific health profiles of individual patients, and syndrome‐specific prophylaxis/diagnosis/monitoring/treatment recommendations developed by the Patient Data Centre, Population Trends Analysis Centre and Biomedical Knowledge Hub (see below), that can be queried by physicians from any component of the health system. It thus constitutes one key defragmentation/cohesion element facilitating greater system efficiency.

#### Patient Data Centre (PDC)

The PDC constitutes a patient data analysis, prevention and treatment optimization centre. Based on determined potential patient susceptibilities and conditions, it personalizes individual patient data profiles in the HC by linking them to best‐practice options for comprehensive diagnosis, disease prevention and therapy, according to latest knowledge provided by the Population Trends Analysis Centre and Biomedical Knowledge Hub (see below), and assists clinicians, where needed. It is staffed by bioinformatic clinicians (generalists/specialists) and informaticians who develop and exploit algorithms for:

- organizing and integrating with individual patient data clinical history, genome sequences, biomarker profiles, microbiome analyses and lifestyle information,
- patient data set analyses to identify susceptibilities, predict future primary/secondary risks, and recommend monitoring regimes and prevention/amelioration/retardation measures,
- Automatic/continuous PAC/BKH‐driven updating of patient profiles and recalibration of symptom‐ and patient‐specific iterative dMC interrogation,
- Analysis of intervention outcome effectiveness indicators.

To avoid ambiguities in patient identities based on names, all patients are assigned a unique identifier, similar to the Orcid persistent digital identifier (Orcid Inc., 2017), coupled to a secure means of matching identifier and patient.

#### Population Trends Analysis Centre (PAC)

The PAC is population‐centric, focusing on epidemiology and trend recognition and analysis, by carrying out aggregate multidimensional meta‐analyses (disease, symptom, metabolic/physiological status, lifestyle, age, genome/biomarker/microbiome, etc.) on HC data. It is responsible for establishing early‐warning systems for disease emergencies and staffed by teams of clinical bioinformaticians, epidemiologists and informaticians, developing and exploiting algorithms for e.g.

- discovery/identification of disease symptom pattern correlations
- stratification of patients and their clinical data into diverse cohorts
- identification of emerging disease trends and appropriate preventive measures
- discovery of new disease syndromes
- assessment of hypothesis‐driven queries
- interfacing with international organizations to share data, predict emergencies and develop rapid responses

#### Biomedical Knowledge Hub (BKH)

The BKH is the interface between the different NCIC components and emerging validated biomedical discoveries. Its primary responsibility lies in prospective/predictive medicine. The BKH (i) evolves novel research designs/strategies, (ii) exploits artificial intelligence for suggesting prospective prophylactic/treatment pathways, (iii) develops intervention outcome effectiveness indicators, and (iv) integrates clinical knowledge with new biomedical research advances.

In this context, one of the most dynamic sectors of medical research in which significant discoveries are being made that will lead to diverse new diagnostic, monitoring, prevention and therapy procedures is microbiology, as documented in this issue of *Microbial Biotechnology*. Examples of recent advances in microbial technologies with implications for clinical settings and applications include:

- microbial biosensors for diagnosis, monitoring and epidemiology (Chang *et al*., 2017),
- nutritional therapy for persistent diarrhoea (Sarker *et al*., 2017),
- new therapeutic strategies for treatment of infections caused by drug‐resistant microbes (Brüssow, 2017; Wong and Santiago, 2017) and recurrent infections by persister cells and biofilms (Wood, 2017),
- the use of designed bacteria to deliver therapeutics (Alvarez and Fernandez, 2017),
- use of metal‐based antimicrobials (Turner, 2017),
- microbiome therapies (Cryan, 2017; O'Toole and Paoli, 2017; Osman, 2017),
- use of synbiotics (synergistic combinations of prebiotics and probiotics) in prevention and therapy (Gurry, 2017),
- tumour‐targeting bacteria‐based cancer therapies (Felgner *et al*., 2017), and
- the use of microbial treatment of clinical environment surfaces to reduce drug‐resistant pathogen burdens (Caselli, 2017)

The BKH is thus a crucial motor of medical advancement and its translation into clinical practice. It will also counteract the current trend in expertise fragmentation and lead to more holistic assessments of patient symptoms, and result in the replacement of linear sequential trial‐and‐error decision tree treatment schedules, based on patient responses to conventional practice drugs, by treatment schedules based on comprehensive exploration of the multidimensional space of personalized medicine that considers patient genomics/microbiome/physiology/health status/lifestyle, and all available intervention options and their predicted consequences for different individuals.

The BKH establishes a robust system of strategic, needs‐based prioritization and formulation of hypotheses for, and design of, clinical trials, and continuously evaluates and proposes updated best‐practice guidelines. It supports development of the parameters of the new patient data documentation system, develops a robust and continuously evolving system of data security, defines quality and information granularity standards for data and certifies potential new data streams into the Healthcare Cloud to prevent its population with inadequate data. It provides both *ad hoc* on‐demand and comprehensive annual health reports to support regulators and health policymakers and makes regular strategic recommendations based on clinical needs. The BKH will become a main pillar of stewardship of the healthcare system, and the foundation of medical student education and knowledge updates for practicing physicians. It would also adopt a leading role in providing medical knowledge to the general populace and hence promote patient medical literacy.

The diverse streams of information (ideally including those from international public health institutions) into the NCIC collectively constitute the Healthcare Data–Biomedical Knowledge Highway Network (Fig. 1, right‐hand panel). This highway aliments the work of the NCIC and is a major driving force for innovation and cooperation.

#### Health Policy Innovation and Evaluation Centre (HPC)

The HPC is the health policy research and design centre expediting the procedure of integrating new research evidence/findings and community needs/preferences and their translation into new policy (see e.g. Fielding and Briss, 2006). It receives and processes regular and special reports with the newest research findings pertinent to health policy from the NCIC and other analysis centres (e.g. think tanks). Its responsibilities include *inter alia*,

- evaluation of existing and development of novel health policy/regulation recommendations in relation to (new) epidemiological trends, medical needs, biomedical discoveries, health technologies, advances on current treatment practices, etc., and
- specification of adequate benchmarking indicators for automatic and continual policy evaluation and rapid review, and of appropriate patient, provider and payer incentives for achieving set healthcare objectives.

## The dMC's role in reducing inequity and costs, and increasing efficiency and quality, of service delivery in healthcare systems

As alluded to in the introduction, over the past decades, considerable effort has been invested in controlling healthcare costs and, in parallel, improving service performance and quality. Healthcare system organization can vary significantly from country to country, but in all countries healthcare resources are limited (OECD, 2015b). A variety of mechanisms exist to incentivize frugal healthcare resource consumption by patients and, more importantly, service provision by healthcare providers, while preserving/improving coverage and quality of services. The most prominent mechanisms adopted in many high‐income countries are (i) payer–provider managed care arrangements with preferred providers and/or insurance ownership of the provider, i.e. discounted services for a specific insuree/patient pool against a pre‐specified healthcare services basket, and (ii) financial risk‐sharing arrangements, i.e. budget responsibility, in combination with periodic performance and quality targets and monitoring (Kutzin, 2001).

A key challenge for achieving equity and increasing efficiency in healthcare markets (in particular in competitive variants) is risk‐selection. If not appropriately dealt with, insurances and providers have the incentive to invest resources in determining and attracting good risks (healthier patients with a higher probability of remaining healthy = less costly) to maximize savings and/or profits. This behaviour can lead to increasingly large pools of uninsured “bad risk” individuals, and thus inequity in access to health care. To disincentivize this behaviour and level the playing field (i.e. reduce overfunding and, more importantly, underfunding), funding is in many cases risk‐adjusted, i.e. weighted according to geo‐, socio‐demographic and health status indicators and predictors (risk adjusters), classifying patients into needs‐based risks to determine the collective risk and thus expenditures of a pool. Insurance funds/providers serving pools with, in relative terms, worse (better) risks receive a higher (lower) proportion of funding to more accurately reflect their prospective expenditure patterns. However, risk adjustment is only as accurate as the explanatory power of the risk adjusters employed and usually considerable uncertainty remains concerning prospective consumption (see e.g. Beck, 2000; Goulão and Perelman, 2014; Kutzin, 2001).

The dMC is a new generation of service provider. It represents, in line with the currently omnipresent trend of automation, a logical step in the evolution of healthcare provision which addresses the most pressing challenges leading to current efforts to tightly manage limited resources while improving access, performance and quality of care. *De facto*, there is no supply‐side risk selection, as the dMC services do not discriminate between different patients (risks), and there exists no incentive for dMCs to over‐ or underprovide services, as the level of services to provide to each dMC patient is determined by algorithms, which are predicated on the most current best‐practice diagnosis/procedure and quality guidelines, and crucially, centrally regulated by the NCIC. Overprovision could be initiated by a small number of regional eClinicians who are, however, also supported by the NCIC, so there exists virtually no incentive for systematic overprovision on part of the dMC‐eCC tandem. Moreover, with the implementation of the proposed dMC‐ and NCIC‐driven individual and population data collection and analytical capabilities, identification of further, more accurate risk adjusters (see above), would be expedited and, in combination with genome, microbiome and biomarker analyses, could potentially lead to virtually individual‐based predictable health intervention needs and outcomes, which is highly complementary to the current and future personalized medicine trend. In addition to the current uncertainty in patient utilization patterns, providers are not equally efficient at delivering services, and therefore, significant, usually unobservable, supply‐side (expenditure‐)uncertainty must be taken into consideration in current risk‐adjustment schemes (see e.g. Newhouse, 1996; Rice and Smith, 2001). As the uncertainty in risk adjustment concerning the variability of service provision efficiency is virtually non‐existent, due to the NCIC‐determined standardization of (i) clinical validation of dMC smart machines/instruments and (ii) routine clinical diagnoses and procedures across all dMCs, dMC‐based healthcare service expenditure variability instead will be primarily based on predictable costs for the operation (overhead) of the dMCs, and the considerably more accurate predictability of patient utilization and expenditure patterns.

Risk‐adjusted capitated payment, a prospective payment per head (patient) based on their predicted utilization needs for a given healthcare services basket within a specified period, has been successful in transferring financial risk (budget responsibility) from payers to healthcare providers with a view, as mentioned above, to control costs and achieve improved performance and quality. Nonetheless, as also discussed above, the accuracy of risk adjustment is limited to predictions of utilization patterns of patient classes, not individuals, so considerable uncertainty remains (see e.g. Emery *et al*., 1997; Rice and Smith, 1999, 2001). Assuming, for example, patients can visit any dMC nationwide and there exists one dMC‐eCC resource pool to which insurance funds and/or individuals contribute, a prospective payment method similar to risk‐adjusted capitation might be well suited to the dMC‐eCC tandem: *per capita risk‐adjusted dMC visit credit*. This credit per patient would be based on an increasingly sophisticated risk‐adjustment scheme facilitated by the above‐mentioned dMC‐NCIC data analyses and feed into a function of patient‐specific predicted level of routine services needed in primary care visits, consisting of e.g.:

- regular check‐ups and prevention measures +
- necessary condition‐specific visits (post‐treatment/hospitalization follow‐ups, monitoring, etc.) +
- prescription renewal (e.g. exchangeable across dMC pharmacies, dispensing physicians or regular pharmacies) +
- X additional visits for unpredictable contingency purposes (infections, minor accidents, etc.).

There does exist the possibility of some patients unnecessarily (over‐)utilizing the dMCs and/or eCCs, i.e. moral hazard^2^ (see e.g. Goulão and Perelman, 2014). However, utilization of behavioural economics, e.g. through informing patients who overutilize the dMCs of the average visit frequency and cost of other dMC users with similar health needs (social norms), would help reduce the potential for moral hazard (see e.g. Cialdini *et al*., 2006).

## The dMC‐NCIC innovation and efficiency axis for integrative healthcare service evolution

Whereas the dMC+eCC tandem achieves a significant efficiency gain at the patient level, by pooling resources at the point of automated healthcare service delivery, the NCIC as a national coordination centre accomplishes an important efficiency gain at the aggregate level by pooling resources to improve the specificity and effectiveness of prevention/treatment strategies for individuals and the entire population and, importantly, by providing cohesion to/reducing fragmentation in the entire health system.

The dMC will be a pioneering new element of health systems – a gateway for the introduction of new technology and increasing exploitation of IT and AI – and will promote evolution of an increasingly medically literate participating patient. The growing involvement of an empowered patient in personal health care, and increased health awareness and motivation to preserve and improve health status, will inexorably lead to healthier lifestyles and reductions in healthcare and social costs. Together with the NCIC, the dMC will be transformative and accelerate health system‐wide integrative evolutionary change towards greater efficiency and cohesion.

The dMC will become the driver of disease reduction by carrying out an increasing range of monitoring tests and diagnostic procedures, not currently carried out routinely, that detect/predict disease predispositions, and regularly sample and analyse biomarkers of (pre)disease and disease susceptibility, and microbiomes. The dMC will thus spearhead personalized medicine in health systems.

As alluded to above, the dMC will also become an innovation driver for the development of an ever‐expanding range of intuitive, easy‐to‐use diagnostic and monitoring instruments and procedures, and patient‐/patient–nurse‐/patient‐remote eCC clinician‐operated on‐site prevention and therapy procedures. Similarly, the NCIC will be a motor for the discovery of disease causes, patient predispositions, environmental and lifestyle risks (determinants of public health), health trends, etc., and hence specification of new diagnostic, monitoring, prevention and therapy needs. Some of the solutions developed in response to the identification of such needs will be first implemented in the dMCs and thus will involve important innovation synergies between the dMC, NCIC and instrument developers. The dMC‐NCIC will thereby become a powerful innovation axis and thus a major source of pull for clinical medicine and translation of new discoveries into clinical practice.

## Collateral benefits

**Figure 2:**
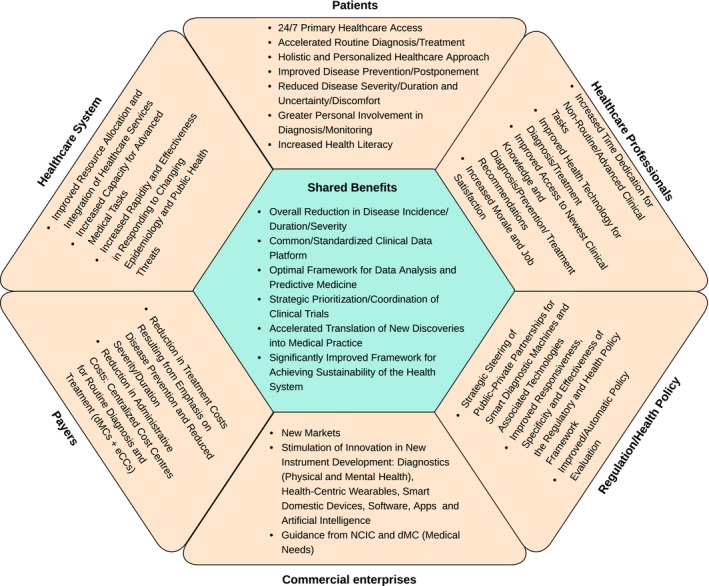
Anticipated Stakeholder Benefits. Benefits primarily for individual stakeholders are shown in yellow, and benefits primarily shared among stakeholders are shown in green.

We predict that implementation of the new health cluster will have significant collateral benefits for stakeholders, some of which are listed in Fig. 2.

In particular, the dMCs and NCIC, and their activities, will be major drivers of innovation and constitute significant new markets for medical instrument and IT developers. This innovation stimulation will accelerate development and deployment of new and improved diagnostic instruments which, in turn, will facilitate new and improve existing health interventions and thus outcomes. For example, the dMCs will provide strong incentives for the development of simple‐to‐use (i) new systems able to deliver novel types of diagnosis, and (2) compacter variants of existing highly sophisticated machines, currently only available in medical centres and operated by skilled technicians (the large markets represented by the dMCs will provide considerable economies of scale, so the new variants will be both patient‐usable and affordable). Other systems of particular interest, all to be linked to the Healthcare Cloud, are as follows: (i) new diagnostic reagents/assays/materials, (ii) smart wearable (Lo *et al*., 2016) and smart daily assistance devices, such as smart walking canes for health parameter monitoring, (iii) wirelessly integrated smart domestic devices, such as fridges, furniture, for the home, workplace and leisure facilities, to e.g. probe and analyse clinically relevant environmental parameters and/or suggest individual dietary options, etc., and (iv) new health software/apps (Boulos *et al*., 2014; Wiederhold, 2015). The data collected from such devices by the Healthcare Cloud would provide invaluable information to the NCIC on individual and population developments, facilitate enhancement of diagnostic/monitoring capabilities, and provide manufacturers with important guidance to generally improve, more precisely predict the effectiveness of, and expand their range of clinical parameter monitoring and other health‐related products. This, in turn, will stimulate advances in and increase the capabilities of the new healthcare cluster, and thereby the entire healthcare system, and facilitate progress towards sustainability.

## Discussion

We present here a concept and roadmap to significantly improve exploitation of informational, technical, financial and human capital healthcare resources. It is designed to considerably reduce/counteract fragmentation and orient health system evolution towards greater strategic cohesion, equity, efficiency and hence sustainability. It is based on the principles of maximization of disease burden reduction, greater exploitation of medical instrument and informatics potentials, purpose‐focused use of healthcare resources, and better exploitation of strategic stakeholder alignment and synergies. In addition to the accelerated exploitation of smart machines, artificial intelligence and growing involvement of an increasingly medically literate, empowered patient, microbial technologies play a key facilitating role in the concept, especially for the (i) lifelong regular sampling and analysis of patient microbiomes, given the mounting evidence for the pervasive implication of microbiome in health and disease, (ii) discovery/creation of metabolite–receptor–ligand–signal transduction systems in microbes for biomarker diagnostics, (iii) use of microbes as cell factories to produce diagnostic reagents, e.g. for lateral flow assays, and, in the foreseeable future, (iv) *ad hoc* drug and vaccine synthesis, etc.

The importance of mental health (and its impact on physical health) requires that it be given equal attention in the strategic planning of healthcare evolution (Lack *et al*., 2015). As physical and mental health services co‐evolve and operational differences reduce, dMCs will be able to provide an expanding range of mental health services. In particular, the dMC‐eCC tandem will provide a number of diagnostic facilities for patients seeking mental healthcare access, including assessment of relevant symptoms and, most importantly, online visual consultation with eCC‐based mental health professionals. At home, wirelessly integrated, e.g., voice‐enabled, smart devices linked to the Healthcare Cloud will play a major role in regularly querying individuals about their physical and mental well‐being and transmitting these data to the Cloud, remind individuals of, e.g., appointments and medication, inform them about new primary and secondary prevention measures (health/lifestyle/immunization/screening) recommended by health authorities, etc. and, most importantly, alert them and the health services when they require medical assistance/consultation. It is becoming increasingly clear that the microbiome, in particular, the gastrointestinal tract microbiota, has a significant and pervasive influence on brain function and mental health (see e.g. Dinan and Cryan, 2017), so the analysis of periodically taken microbiome samples will not only provide new insights into mental disease and its onset, but also indicate potential therapy options involving microbiota transplantations.

While we have presented our concept primarily in the context of high‐income countries, the dMC‐eCC tandem is equally relevant to the low‐ and middle‐income country (LMIC) setting. Initially, it would represent particular value in rural areas, where access to and affordability of healthcare services, and infrastructure consolidation, remain important challenges. As dMCs are scalable and customizable, the provided services can be adapted to any desired level of generic and specific needs, that is (and this applies equally to low‐, middle‐ and high‐income settings) a variety of dMC versions (standard routine services + specific services for catchment area‐specific differential health needs) would be tested and calibrated in pilot studies for different regions and, after initial shortcomings are identified and remedied, would be implemented on a broader scale. Especially in the light of the Grand Convergence discussion (Jamison *et al*., 2013), we see a window of opportunity and unique momentum for the dMCs to stimulate, facilitate and expedite biomedical innovation, catalyse universal healthcare coverage and present a compelling business opportunity for public and private funders in and for LMICs.

Finally, it should be emphasized that the dMCs will complement, not replace, existing healthcare services. It is to be anticipated that the immediacy of access to the services of the dMCs will be perceived as a significant advantage by some, and in particular patients used to interacting with smart devices will feel comfortable with, and increasingly use, the dMCs. We also anticipate that as the spectrum of diagnostic options in dMCs grows, local medical practices will increasingly adopt and/or co‐design/optimize smart machines and procedures used in the dMCs across healthcare providers, to increase their efficiency and synergy effects, so that the distinction between dMCs and traditional medical practices will blur.

## Conflict of Interest

None to declare.

## Notes

1 This is primarily attempted by tightly managing and coordinating care at the provider level, sharing financial risk (e.g. budget responsibility) between payers, healthcare providers and patients, and redistributing funds and financial risks for different pools of more or less healthy, and thus costly, insurees and/or patients (Kutzin, 2001).

2 If an individual is insured against financial risk, he/she might, e.g., engage in riskier behaviour (*ex ante*) or access more (healthcare) services than necessary (*ex post*) (Goulão and Perelman, 2014).
